# Joint Effect of Maternal Tobacco Smoking and Pregestational Diabetes on Preterm Births and Congenital Anomalies: A Population-Based Study in Northern Italy

**DOI:** 10.1155/2018/2782741

**Published:** 2018-06-28

**Authors:** Lucia Borsari, Carlotta Malagoli, Martha M. Werler, Kenneth J. Rothman, Marcella Malavolti, Rossella Rodolfi, Gianfranco De Girolamo, Fausto Nicolini, Marco Vinceti

**Affiliations:** ^1^Department of Biomedical, Metabolic and Neural Sciences, Environmental, Genetic and Nutritional Epidemiology Research Center (CREAGEN), University of Modena and Reggio Emilia, Modena 41125, Italy; ^2^Clinical and Experimental Medicine PhD Program, University of Modena and Reggio Emilia, Modena, Italy; ^3^Department of Epidemiology, Boston University School of Public Health, Boston, MA 02118, USA; ^4^Research Triangle Institute, Research Triangle Park, NC 27709, USA; ^5^Local Health Authority of Reggio Emilia, Reggio Emilia 42122, Italy; ^6^Department of Public Health, Unit of Epidemiology and Risk Communication, Local Health Authority of Modena, Modena 41126, Italy

## Abstract

Smoking and pregestational diabetes (PGD) are recognized risk factors for adverse pregnancy outcomes, but to date, no population-based study has investigated their joint effects. Using hospital discharges, we identified all women with PGD delivering in Emilia-Romagna region during 2007–2010 matched 1 : 5 with parturients without diabetes. Our study endpoints were preterm births and congenital anomalies. We measured interaction between PGD and maternal smoking, by calculating excess prevalence and prevalence ratio due to interaction, relative excess risk due to interaction (RERI), attributable proportion (AP), and the synergy index (S). Analyses were performed in the overall study population and in the subgroup whose PGD was validated through diabetes registers. The study included 992 women with PGD (10.5% smokers) and 4788 comparison women (11.9% smokers). The effects of PGD and maternal tobacco smoking were greater than additive for both preterm birth (excess prevalence due to interaction = 11.7%, excess ratio due to interaction = 1.5, RERI = 2.39, AP = 0.51, S = 2.82) and congenital anomalies (excess prevalence due to interaction = 2.2%, excess ratio due to interaction = 1.3, RERI = 1.33, AP = 0.49, S = 5.03). Joint effect on both endpoints was confirmed in the subgroup whose PGD status was validated. In conclusion, we found that maternal tobacco smoking and PGD intensify each other's effect on preterm birth and congenital anomalies.

## 1. Introduction

Tobacco smoking during pregnancy is one of the main modifiable risk factors associated with adverse maternal and fetal outcomes, including intrauterine growth restriction, ectopic pregnancy, premature birth, and congenital anomalies [1–4]. Pregnancy therefore represents a unique motivation for smoking cessation, and more women quit smoking during pregnancy than at any other time in their lives [5]. However, despite the known risks, the majority of women smoking at conception continue to smoke. More than 10% of women smoke during pregnancy in many high-income countries [6, 7]. In Italy, epidemiologic studies and routinely collected health data show that tobacco smoking prevalence among pregnant population varies from 6.7% to 22.3%. Tobacco smoking is associated with young maternal age and low economic status or education level [8–11].

Pregestational diabetes (PGD) represents the most common chronic condition complicating pregnancy. The number of affected pregnancies has been increasing, largely due to the obesity epidemic and consequent increase in type 2 diabetes in younger women [12, 13]. Several studies showed that PGD is an important risk factor for adverse pregnancy outcomes. In particular, women with existing diabetes have a risk of pregnancy resulting in major structural congenital anomaly up to fourfold greater than women without PGD [14–18]. In addition, the risk of preterm delivery, either spontaneous or indicated, was increased in women with pregnancies complicated by PGD, with studies reporting rates up to sevenfold higher than that among women not affected [19–21].

While maternal smoking and diabetes have been both recognized as independent relevant risk factors for adverse pregnancy outcomes, little is known about their interaction. Nicotine, one of the major bioactive substances in cigarettes, has been shown to alter glucose homeostasis, implying an important role for this agent in the development of cardio-metabolic and other complications in diabetic patients [22]. Moreover, tobacco smoking seems able to amplify the effect of some risk factors on outcomes such as cardiovascular mortality or diseases, disability, and gestational diabetes, implying synergism between smoking and other factors [23–27].

The aim of this study was to investigate the joint effect of smoking and PGD on the risk of preterm birth and congenital anomalies in a population-based cohort of Italian pregnant women.

## 2. Materials and Methods

### 2.1. Study Population

Following approval by the Modena Province Ethics Committee, we conducted a retrospective cohort study by identifying all deliveries in Emilia-Romagna region (Northern Italy, population about 4,000,000) in the 2007–2010 period through the Hospital Discharge (HD) database (International Classification of Diseases version 9 Clinical Medicine (ICD-9-CM) codes V27.0-V27.9). We selected parturients with PGD through the presence of ICD-9-CM codes 250.xx or 648.0x listed anywhere on the HD records. For each selected HD record, we matched five comparison mothers without diabetes, according to year of birth and delivery, province of residence, and referral hospital. Among all mothers who matched the index mother by these criteria, we randomly selected the five comparators. To carry out this sampling of matched comparison women, we used the STATA-12 optmatch2 routine. If five women without diabetes were not available to match a woman with diabetes, we accepted a lower number. No matched woman was available for 2.3% of the women with diabetes. For these women, we relaxed the matching criteria first for province of residence, then for hospital of delivery, and finally for year of delivery. Women who experienced more than one delivery during the 4-year study period were included as multiple observations. Twin pregnancies were excluded.

**Figure 1:**
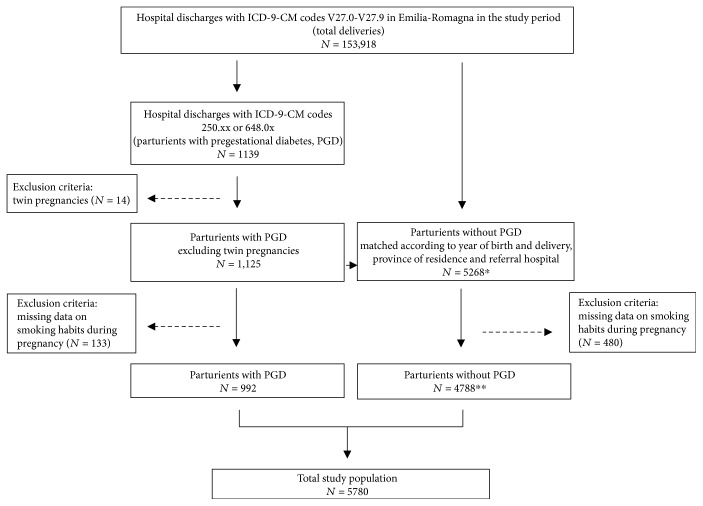
Flowchart of the study population. ^∗^ 954 cases matched 5 controls, 66 cases matched 4 controls, 49 cases matched 3 controls, 31 cases matched 2 controls and 25 cases matched 1 control. ^∗∗^ 921 cases matched 5 controls, 22 cases matched 4 controls, 17 cases matched 3 controls, 12 cases matched 2 controls and 20 cases matched 1 control.

We used the birth certificates to collect information on tobacco smoking habits of the mother, as well as on other sociodemographic and clinical characteristics used as covariates in the analysis (nationality, maternal age at the time of delivery, and education level of both parents). Smoking habits during pregnancy were therefore self-reported by mothers. Maternal smoking was defined as smoking for any period during pregnancy. Thus, it includes both women smoking only in early pregnancy and women smoking throughout pregnancy, regardless of the number of cigarettes smoked. Women who never smoked or who quit smoking before pregnancy were considered nonsmokers. Missing data on smoking habit during pregnancy was considered as an exclusion criterion in the selection of the study population. Method for selecting the study population is shown in Figure 1.

Lastly, through the population-based diabetes registers recently established in two out of nine provinces of the Emilia-Romagna region [28], Modena and Reggio-Emilia, we validated PGD diagnosis for the subgroup of women residing in these two provinces. Diabetes registers are based on case identification algorithms involving both administrative and clinical data, so their data tend to be more complete and accurate than data from other sources [29].

### 2.2. Pregnancy Adverse Outcomes

We focused on two adverse pregnancy outcomes, preterm births and congenital anomalies. We defined preterm birth as gestational age at delivery, according to the birth certificate, of <37 weeks. We detected congenital anomalies by matching each selected birth certificate to the Registry of Birth Defects of the Emilia-Romagna Region named IMER (*Indagine sulle Malformazioni congenite in Emilia Romagna*). This registry, established in 1978, is part of the European Network for the Surveillance of Congenital Anomalies EUROCAT [30].

### 2.3. Data Analysis

We measured the interaction between tobacco smoking and PGD as the excess over additivity of effects, comparing the combined effect of the two exposures with the sum of their individual effects. Firstly, we computed prevalence difference (PD) and prevalence ratio (PR) with associated 95% confidence interval (CI) of preterm birth and congenital anomalies using Episheet statistical software (version of April 10, 2017, downloaded from http://krothman.org/Episheet.xls). Women without PGD and nonsmokers represented the reference category. Excess prevalence of preterm birth and congenital anomalies due to interaction was calculated as the difference between PD in women exposed to both smoking and PGD (PD_11_) and the sum of PD in women exposed to only smoking (PD_01_) or only PGD (PD_10_) (excess prevalence due to interaction = PD_11_ − (PD_01_ + PD_10_)). Excess ratio due to interaction was calculated as PR_11_ − PR_01_ − PR_10_ + 1 [31].

Then, we measured departures from additivity using appropriately defined relative measures of effect derived from multiplicative regression models. We estimated relative risks (RRs) from the odds ratios generated by logistic regression models, introducing PGD and maternal smoking in the models as a product term. In this model, we included maternal age at delivery, nationality, and parents' education as confounding variables. We used the estimates from logistic regression models to calculate the relative excess risk due to interaction (RERI), the attributable proportion due to interaction (AP), and the synergy index (S), using an Excel spreadsheet by Andersson et al. [32]. RERI should be interpreted as the relative excess risk (RR-1) in those with both exposures minus that expected on the basis of the sum of the relative excess risks for the individual exposures. The AP represents the proportion of disease that is due to interaction among persons with both exposures. The synergy index S measures the extent to which the joint effect for both factors together, expressed as the relative excess risk, which exceeds the sum of the relative excess risk for each of the two factors' separate effect [33, 34].

Statistical analyses were performed by using Stata 15 statistical software (StataCorp LP, College Station, TX).

## 3. Results

The study included 5780 pregnant women, among them 992 with PGD and 4788 without PGD (Figure 1). The prevalence of tobacco smoking was similar among women with and without PGD (10.5% and 11.9%, resp.). General characteristics of the study population are presented in Table 1. Overall, we identified 536 (9.3%) preterm births and 118 (2.1%) newborns with one or more congenital anomalies.

Prevalence differences, PRs and RRs of preterm birth and congenital anomalies are reported in Table 2. We used these results to calculate measures of interaction between PGD and tobacco smoking, and we found that the effects were greater than additive for both preterm birth and congenital anomalies. The excess prevalence of preterm birth due to interaction was 11.7%, and the excess ratio of preterm birth due to interaction was 1.5. The joint effects on preterm birth were confirmed also when confounding variables were considered, with RERI = 2.39 (95% CI: 0.25, 4.53), AP = 0.51 (95% CI: 0.26, 0.75), and S = 2.82 (95% CI: 1.39, 5.79). An AP of 0.51 for preterm birth means that we estimate that 51% of the preterm births occurring to women who had diabetes and were smokers is attributable to the interaction between these two risk factors, assuming all the effects are causal and biases are controlled. As for congenital anomalies, we found excess prevalence due to interaction = 2.2%, excess ratio due to interaction = 1.3, RERI = 1.33 (95% CI: −1.19, 3.88), AP = 0.49 (95% CI: 0, 1.05), and S = 5.03 (95% CI: 0.23, 42.25). The AP of 0.49 indicates again that about half of the occurrence of births with congenital anomalies can be attributed to the interaction of tobacco smoking with pregestational diabetes.

Effect estimates for major categories of congenital anomalies were statistically unstable because of the small number of subjects. The congenital anomalies for which prevalence was considerably increased among diabetic women who also smoked during pregnancy were those affecting the cardiovascular system (RR = 2.88, 95% CI: 0.67–12.37), the genitourinary system (RR = 3.46, 95% CI: 0.43–27.62), and cleft palate/lip (RR = 7.42, 95% CI: 0.85–64.71), with RERI = 2.70 (95% CI: −1.52–6.91), 0.58 (95% CI: −6.88–8.05), and 4.96 (95% CI: −10.73–20.64), respectively. Concerning only anomalies of the cardiovascular system, which accounted for the highest number of congenital anomalies, we assessed the single anomalies, and we found that the increased risk in the offspring of women both smoking and affected by diabetes involved mainly atrial septal defects (RR = 9.29, 95% CI: 1.04–83.13), with RERI = 8.02 (95% CI: −12.05–28.09).

The analysis restricted to the subgroup of women validated through provincial diabetes registers of Modena and Reggio-Emilia included 108 women with PGD and 513 comparison women. Prevalence differences, PRs and RRs of preterm birth, and congenital anomalies among these women are reported in Table 3. Patterns were similar among these validated cases, but subject to more variation as a consequence of the small number of validated cases (for preterm birth, excess prevalence due to interaction = 5.3%, excess ratio due to interaction = 0.6, RERI = 1.75 (95% CI: −7.14, 10.63), AP = 0.29 (95% CI: 0, 1.37), S = 1.54 (95% CI: 0.24, 9.75) and for congenital anomalies, excess prevalence due to interaction = 3%, excess ratio due to interaction = 2.2, RERI = 5.44 (95% CI: −23.03, 33.91), AP = 0.42 (95% CI: 0, 1.75), S = 1.84 (95% CI: 0.14, 21.19)).

## 4. Discussion

This is the first population-based study evaluating the interaction between diabetes and tobacco smoking in the specific population of pregnant women. Among women with both PGD and smoking, we found 11 preterm births and 2 congenital anomalies per 100 newborns more than we would expect on the basis of the sum of the separate effects of PGD and smoking, consistent with the presence of causal interaction between the two factors. In particular, calculation of AP showed that 51% of preterm births and 49% of congenital anomalies that occurred in the offspring of women with PGD and tobacco smoking habit were attributable to the interaction of the two exposures. This excess risk over additivity of effects was confirmed in the analysis performed on the subpopulation validated through the provincial diabetes registers. Despite the limited statistical stability from small numbers in this subgroup, the analysis of the provincial Diabetes Registers is reassuring, considering the higher accuracy in identifying cases of diabetes mellitus in this data source. Indeed, two Canadian and American studies [35, 36] have already highlighted that the use of only administrative data to identify diabetes in populations such as pregnant women could involve substantial misclassifications, mainly due to a limited ability to distinguish between pregestational and gestational diabetes.

These results are consistent with the hypothesis that PGD has effects that make offspring of affected women more susceptible to the effects of tobacco smoke. The negative effect of tobacco smoking on glycemic control, which has been already demonstrated [37], could partially explain the joint effect between the two risk factors. In fact, poor glycemic control at preconception or during pregnancy has been identified as one of the main factors associated with adverse pregnancy outcomes in women with diabetes [37–39].

Among the limitations of our study are considerable statistical variability of some estimates and lack of information about the metabolic status of the diabetic women. In addition, there are the possible confounding effects of concomitant obesity, folic acid consumption, and drug prescription at the time of pregnancy. We could not retrieve information about these variables for both parturients with and without diabetes. In addition, some exposure misclassification may have occurred for tobacco smoking during pregnancy, because of possible underreporting in studies relying on self-report [40]. Lack of availability of individual data, however, is frequently an inherent limitation of studies based on administrative or registry databases like the present one. On the other hand, administrative data affords much larger study sizes and may reduce selection bias. Lastly, given the high prevalence of preterm births in the doubly exposed group, the adjusted odds ratios derived from the logistic regression are slight overestimates of the prevalence ratios.

These results have greater importance because the prevalence of tobacco smoking during pregnancy remains high, as we found here, and was also reported in previous studies [8–11]. This is the first study reporting the prevalence of tobacco smoking among Italian pregnant women with PGD, and we unexpectedly found that it was similar to that found among pregnant women without PGD (more than 10%). This finding is concerning in view of the adverse risks related to diabetes and more so as a result of the interaction between the two factors.

## 5. Conclusions

In conclusion, our results indicate an interaction between maternal tobacco smoking and pregestational diabetes in increasing the risk of preterm birth and congenital anomalies. Given the still high prevalence of smokers among pregnant women, including those with PGD, and the interactive relation with PGD, further efforts to discourage smoking during pregnancy among women with diabetes would have a considerable public-health benefit. Further studies, including larger samples of pregnant women, would help to clarify the extent of this benefit as well as to deeply understand the relation between smoking and diabetes on adverse pregnancy outcomes.

## Figures and Tables

**Table 1:** Demographic characteristics of the study population, according to maternal pregestational diabetes and smoking habits.

	Women with PGD					Women without PGD				
	Nonsmoking		Smoking		Total	Nonsmoking		Smoking		Total
	*n*	(%)	*n*	(%)	*n*	*n*	(%)	*n*	(%)	*n*
*Age at delivery*										
<35 years	502	(56.5)	68	(65.4)	**570**	2469	(58.5)	337	(59.1)	**2806**
≥35 years	386	(43.5)	36	(34.6)	**422**	1749	(41.5)	233	(40.9)	**1982**
*Nationality*										
Italian	465	(52.4)	89	(85.6)	**554**	3157	(74.8)	511	(89.6)	**3668**
Other	423	(47.6)	15	(14.4)	**438**	1061	(25.2)	59	(10.4)	**1120**
*Education*										
Elementary/middle school	383	(43.1)	42	(40.4)	**425**	1129	(26.8)	181	(31.8)	**1310**
High school	360	(40.5)	42	(40.4)	**402**	1883	(44.6)	288	(50.5)	**2171**
University	145	(16.3)	20	(19.2)	**165**	1206	(28.6)	101	(17.7)	**1307**
*Father's education*										
Elementary/middle school	423	(47.6)	49	(47.1)	**472**	1362	(32.3)	236	(41.4)	**1598**
High school	314	(35.4)	44	(42.3)	**358**	1734	(41.1)	240	(42.1)	**1974**
University	110	(12.4)	10	(9.6)	**120**	903	(21.4)	72	(12.6)	**975**
Missing	41	(4.6)	1	(1.0)	**42**	219	(5.2)	22	(3.9)	**241**
*Total*	888	(100.0)	104	(100.0)	**992**	4218	(100.0)	570	(100.0)	**4788**

PGD = pregestational diabetes.

**Table 2:** Prevalence difference per 100, prevalence ratio and relative risk of preterm birth, and congenital anomalies associated with pregestational diabetes and/or tobacco smoking, Emilia-Romagna region, 2007–2010.

PGD	Smoking	Total	*n*	(%)	PD^∗^100	(95% CI)	PR	(95% CI)	RR^∗^	(95% CI)
			Preterm birth							
No	No	4218	317	(7.51)	Ref.		Ref.		Ref.	
No	Yes	570	39	(6.84)	−0.67	(−2.89, 1.54)	0.91	(0.66, 1.25)	0.89	(0.63, 1.27)
Yes	No	888	151	(17.01)	9.48	(6.89, 12.08)	2.26	(1.89, 2.70)	2.41	(1.93, 3.02)
Yes	Yes	104	29	(27.88)	20.36	(11.67, 29.06)	3.71	(2.67, 5.15)	4.69	(2.97, 7.41)

			Congenital anomalies							
No	No	4218	79	(1.87)	Ref.		Ref.		Ref.	
No	Yes	570	10	(1.75)	−0.12	(−1.27, 1.03)	0.93	(0.49, 1.79)	0.92	(0.47, 1.79)
Yes	No	888	24	(2.70)	0.83	(−0.31, 1.97)	1.44	(0.92, 2.26)	1.41	(0.88, 2.25)
Yes	Yes	104	5	(4.81)	2.93	(−1.22, 7.08)	2.57	(1.06, 6.23)	2.66	(1.05, 6.71)

^∗^Adjusted for maternal age at delivery, nationality, and parents' education level. PGD = pregestational diabetes; PD = prevalence difference; PR = prevalence ratio; RR = relative risk.

**Table 3:** Prevalence difference per 100, prevalence ratio and relative risk of preterm birth, and congenital anomalies associated with pregestational diabetes and/or tobacco smoking, Provinces of Modena and Reggio Emilia, 2007–2010.

PGD	Smoking	Total	*n*	(%)	PD^∗^100	(95% CI)	PR	(95% CI)	RR	(95% CI)
			Preterm birth							
No	No	449	40	(8.91)	Ref.		Ref.		Ref.	
No	Yes	64	7	(10.94)	2.02	(−6.12, 10.17)	1.23	(0.57, 2.64)	1.30	(0.55, 3.08)
Yes	No	100	30	(30.01)	21.09	(11.68, 30.49)	3.37	(2.21, 5.14)	3.94	(2.25, 6.92)
Yes	Yes	8	3	(37.50)	28.6	(−7.37, 64.55)	4.21	(1.55, 11.51)	5.99	(1.37, 26.29)

			Congenital anomalies							
No	No	449	6	(1.34)	Ref.		Ref.		Ref.	
No	Yes	64	3	(4.69)	3.35	(−1.97, 8.67)	3.51	(0.89, 13.81)	4.46	(1.04, 19.09)
Yes	No	100	6	(6.01)	4.66	(−0.13, 9.46)	4.49	(1.47, 13.71)	4.01	(1.22, 13.18)
Yes	Yes	8	1	(12.50)	11.16	(−13.36, 35.68)	9.35	(1.13, 77.62)	12.91	(1.30, 127.75)

^∗^Adjusted for maternal age at delivery, nationality, and parents' education level. PGD = pregestational diabetes; PD = prevalence difference; PR = prevalence ratio; RR = relative risk.

## Data Availability

The datasets generated during and/or analyzed during the current study are not publicly available due to the personal data protection Italian rules and were accessed by the study authors following a specific application to the Modena Province Ethics Committee (Protocol note 3188/11). They are however available on an aggregate basis from the corresponding author.

## Abbreviations

PGD: Pregestational diabetes
HD: Hospital discharge
ICD: International Classification of Diseases
IMER: Indagine sulle Malformazioni congenite in Emilia Romagna (Registry of Birth Defects of the Emilia-Romagna Region)
PD: Prevalence difference
PR: Prevalence ratio
RR: Relative risk
RERI: Relative excess risk due to interaction
AP: Attributable proportion due to interaction
S: Synergy index.
